# Supplementary material for: High Prevalence of Myocardial Bridging Detected in an Indonesian Population Using Multi-Detector Computed Tomography
Source: Medicina (Kaunas). 2024 May 10;60(5):794. doi: 10.3390/medicina60050794 (PMC11123036; doi:10.3390/medicina60050794)
Supplement: Supplementary file 1 [file medicina-60-00794-s001.zip › medicina-2983525-supplementary.pdf]

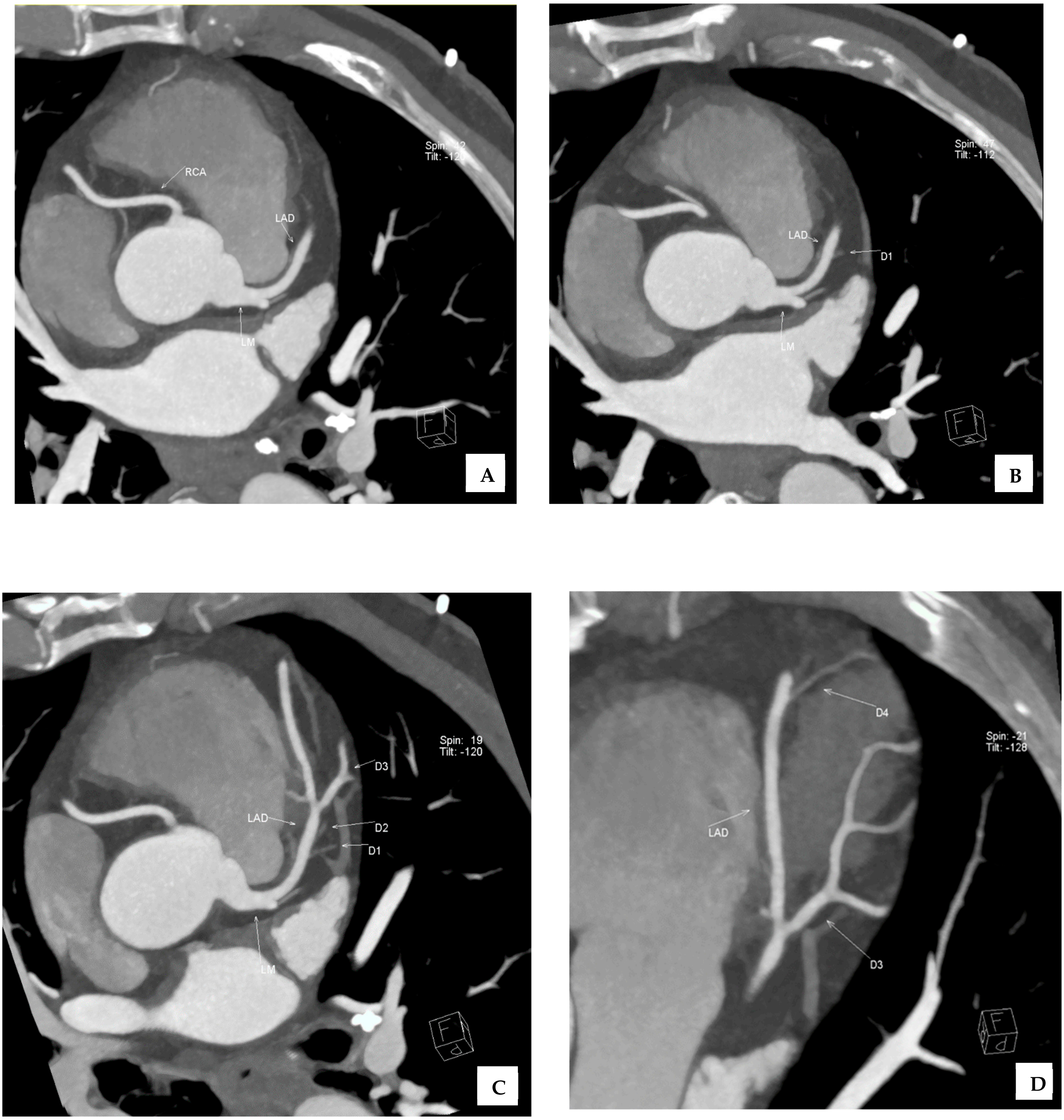
RCA
LAD
LM
A
LAD
D1
LM
Tilt: -112
B
D3
LAD
D2
D1
LM
Spin: 19
Tilt: -120
C
D4
LAD
D3
Spin: -21
Tilt: -128
D

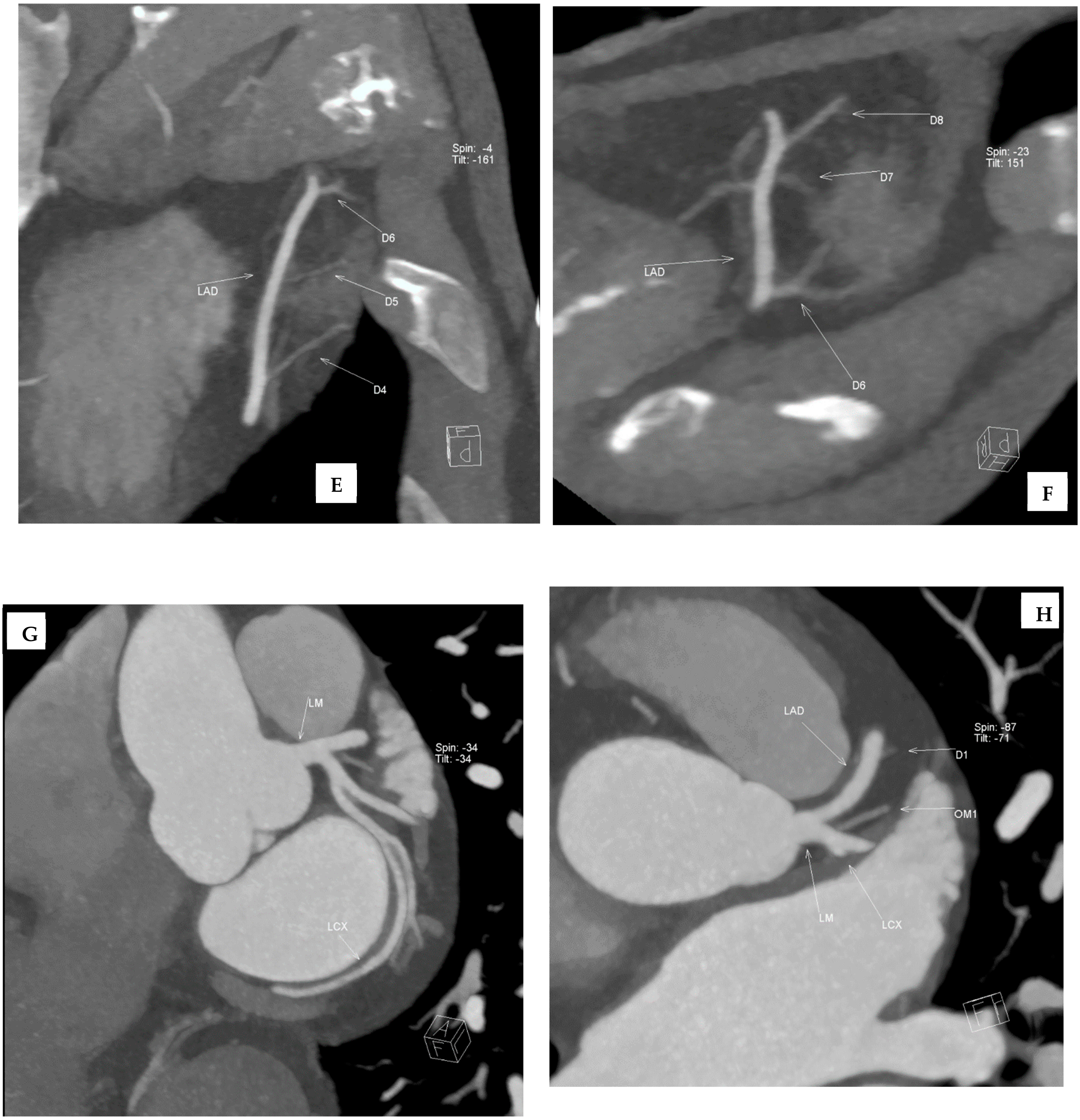
E
LAD
D6
D5
D4
Spin: -4
Tilt: -161
F
LAD
D8
D7
D6
Spin: -23
Tilt: 151
G
LM
LCX
Spin: -34
Tilt: -34
H
LAD
D1
OM1
LM
LCX
Spin: -87
Tilt: -71

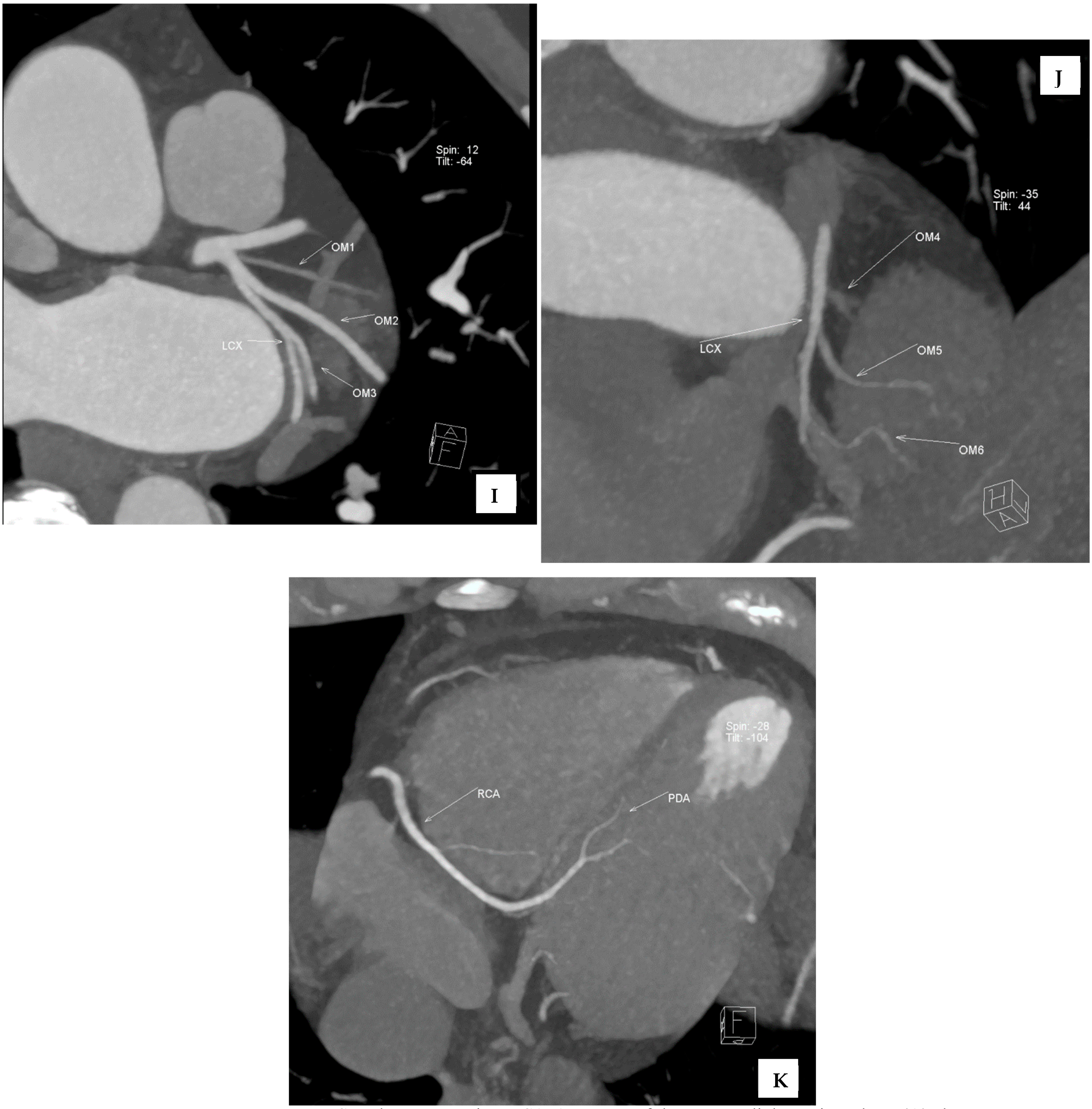

Supplementary Figure S1. Anatomy of the myocardial arteries where (A) shows the right coronary artery (RCA), the left anterior descending (LAD) and left marginal artery (LM); (B) The first diagonal branch of LAD (D1); (C) Shows the D1, second diagonal branch (D2) as well as the third diagonal branch of LAD (D3); (D) This figure shows the fourth diagonal branch of LAD (D4); (E) Shows the fifth diagonal branch (D5) and the sixth diagonal branch of LAD (D6); (F) Shows the seventh diagonal branch (D7) and the eight diagonal branch of LAD (D8); (G) The left marginal (LM) and left circumflex artery (LCX) are shown here; (H) The first obtuse marginal artery (OM1) can be visualized here along with LCX, LM, LAD, and D1; (I) This figure shows the second obtuse marginal artery (OM2) and the third obtuse marginal artery (OM3); (J) The fourth obtuse marginal artery (OM4), the fifth obtuse marginal artery (OM5), and the sixth obtuse marginal artery (OM6) are visualized here along with LCX; and (K) the posterior descending artery (PDA) can be visualized here
